# Molecular and Cellular Mechanisms of Electronegative Lipoproteins in Cardiovascular Diseases

**DOI:** 10.3390/biomedicines8120550

**Published:** 2020-11-29

**Authors:** Liang-Yin Ke, Shi Hui Law, Vineet Kumar Mishra, Farzana Parveen, Hua-Chen Chan, Ye-Hsu Lu, Chih-Sheng Chu

**Affiliations:** 1 Department of Medical Laboratory Science and Biotechnology, College of Health Sciences, Kaohsiung Medical University, Kaohsiung 807378, Taiwan; kly@gap.kmu.edu.tw (L.-Y.K.); shlaw9994@gmail.com (S.H.L.); vineetkmishra.jh@gmail.com (V.K.M.); fparveen.jh@gmail.com (F.P.); 2 Graduate Institute of Medicine, College of Medicine and Drug Development and Value Creation Research Center, Kaohsiung Medical University, Kaohsiung 807378, Taiwan; 3 Center for Lipid Biosciences, Kaohsiung Medical University Hospital, Kaohsiung Medical University, Kaohsiung 807377, Taiwan; huachen.chan@gmail.com (H.-C.C.); yehslu@cc.kmu.edu.tw (Y.-H.L.); 4 Division of Cardiology, Department of International Medicine, Kaohsiung Medical University Hospital, Kaohsiung 807377, Taiwan; 5 Division of Cardiology, Department of Internal Medicine, Kaohsiung Municipal Ta-Tung Hospital, Kaohsiung 80145, Taiwan

**Keywords:** electronegative LDL, LDL(−), L5 LDL, oxidized LDL, oxLDL, lectin-like oxLDL receptor-1, LOX-1, dyslipidemia, endothelial dysfunction, atherosclerosis, cardiovascular disease

## Abstract

Dysregulation of glucose and lipid metabolism increases plasma levels of lipoproteins and triglycerides, resulting in vascular endothelial damage. Remarkably, the oxidation of lipid and lipoprotein particles generates electronegative lipoproteins that mediate cellular deterioration of atherosclerosis. In this review, we examined the core of atherosclerotic plaque, which is enriched by byproducts of lipid metabolism and lipoproteins, such as oxidized low-density lipoproteins (oxLDL) and electronegative subfraction of LDL (LDL(−)). We also summarized the chemical properties, receptors, and molecular mechanisms of LDL(−). In combination with other well-known markers of inflammation, namely metabolic diseases, we concluded that LDL(−) can be used as a novel prognostic tool for these lipid disorders. In addition, through understanding the underlying pathophysiological molecular routes for endothelial dysfunction and inflammation, we may reassess current therapeutics and might gain a new direction to treat atherosclerotic cardiovascular diseases, mainly targeting LDL(−) clearance.

## 1. Introduction

Approximately 1.9 billion people are obese or overweight worldwide [1]. Obesity is associated with excessive calorific intake and microvasculature damage, resulting in atherosclerosis, diabetes, and cardiovascular diseases (CVDs) [2]. The prevalence of CVDs has significantly increased in the past few decades [3]. Current strategies against CVDs mainly focus on lowering the level of low-density lipoprotein cholesterol (LDL-C) [4,5]. Intensive-dose statin therapy has been endorsed for clinical atherosclerotic vascular disease (ASCVD); however, it also increases statin-related side effects and intolerance [6,7]. To figure out this dilemma and find a balanced solution, here we address the mechanistic players behind these metabolic disturbances through the following disease progression steps: unhealthy lifestyle and unbalanced diet lead to obesity, chronic inflammation, and development of atherosclerosis and CVDs [8,9,10].

**Figure 1:**
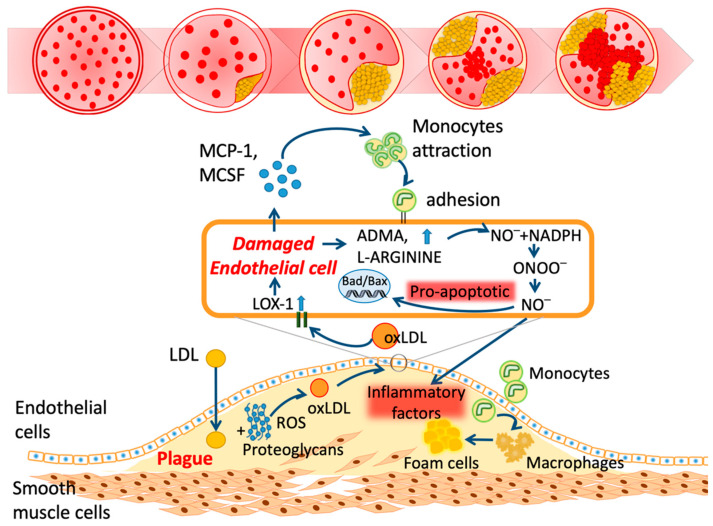
Schematic mechanism of atherosclerosis. LDL: low-density lipoprotein; ROS: reactive oxygen species; oxLDL: oxidized LDL; LOX-1: lectin-like oxidized LDL receptor-1; ADMA: asymmetric dimethylarginine; NO: nitric oxide; NADPH: nicotinamide adenine dinucleotide phosphate; ONOO: peroxynitrite; Bad: BCL2-associated agonist of cell death; Bax: Bcl-2-associated X protein; MCP-1: monocyte chemoattractant protein-1; MCSF: macrophage colony-stimulating factor.

The onset of atherosclerosis initiates vascular lipid deposition, luminal narrowing, and plaque expansion. Unstable plaque deposits further lead to myocardial infarction and stroke [11]. Plaque consists of LDL-C variants, lipids, leukocytes, and inflammasomes in the vascular walls (Figure 1) [11,12]. In addition, several mediators of vasoconstriction, platelet aggregation, inflammatory chemokines, leukocyte adherence, and nitric oxide (NO) disturb the endothelial homeostasis [13]. LDL variants such as oxidized LDL (oxLDL) are essential constituents in the pathogenesis of atherosclerosis and CVDs [14,15,16]. Differing from the in vitro preparation of oxLDL, electronegative LDL (LDL(−)) is separated from human plasma using fast-protein liquid chromatography equipped with an anion exchange column [17]. According to the physical properties of LDL(−), it can be defined as the minimized oxLDL [18,19].

Accumulating evidence shows that LDL(−) could be a novel marker for ASCVD, and levels of LDL(−) are positively correlated with the increasing severity of CVDs [20,21,22]. LDL(−) serves as a pivotal target for further studies and clinical development strategies beyond statins therapies. By targeting LDL(−), we summarize its pathophysiological links and highlight the molecular mechanisms of atherogenic lipids in the current review.

## 2. Properties of Electronegative Low-Density Lipoprotein (LDL(−))

### 2.1. Chemical Properties of LDL(−)

LDL(−) differs from LDL(+) in many aspects [23]. Regarding the lipid components, LDL(−) contains higher concentrations of triglycerides, non-esterified fatty acids (NEFA), lysophosphatidylcholine (LPC), platelet-activating factor (PAF), and ceramide [24,25,26,27]. Notably, lipid extracts of LDL(−) contribute to the atherogenic effects on endothelial cells and immune cells [27,28]. Regarding its protein composition, LDL(−) shows additional proteins such as apolipoprotein AI (apoAI), apolipoprotein E (apoE), and apolipoprotein CIII (apoCIII) [29]. Furthermore, the conformation of apoB100 in LDL(−) is altered and has higher competency to bind with proteoglycans [30,31,32]. Based on the sodium chloride gradient, Chen et al. successfully divided LDL into five subfractions, L1–L5, with increasing electronegativity [29,33,34]. L1 LDL is unmodified; in contrast, L5 LDL is highly *O*-glycosylated on the apoB100 and apoE [28,35]. The terminal glycan of apoE glycosylation (94S, 194T, 289T) in L5 LDL is sialic acid. This sialic-acid-containing glycan increases the electronegativity and hydrophilicity [35]. However, by dividing human plasma LDL into either two subfractions ((+) and (−)) or five (L1–L5), the most electronegative subfractions show similar properties and apoptotic effects on endothelial cells. Thus, we will be using LDL(−) throughout this review.

### 2.2. Receptors of LDL(−)

LDL(−) is not recognized by the LDL receptor. Instead, it goes through lectin-like oxLDL receptor-1 (LOX-1), which is highly expressed in endothelial cells, immune cells, platelets, and adipocytes [36,37,38,39]. Transfection with LOX-1-specific small interfering RNAs (si*LOX-1*) to endothelial cells may attenuate LDL(−)-induced downstream signaling [36]. LOX-1-neutralizing antibodies such as TS20 (for bovine) [40], TS58 (for mouse) [41], and TS92 (for human) [42,43] can inhibit the internalization of LDL(−). Genetic knockout *LOX-1* also protects against the harmful effects of LDL(−) [37,38]. Higher content of PAF on LDL(−) activates the PAF receptor (PAFR) and leads to endothelial cell apoptosis [33]. Incubating PAF acetylhydrolase (PAF-AH) with LDL(−) or pretreatment of WEB-2086 attenuates LDL(−)-induced apoptosis [33]. In addition, ceramide-rich LDL(−) activates toll-like receptor 4 (TLR4) and the cluster of differentiation 14 (CD14) on monocytes that results in cytokine release. Using the TLR4 inhibitor, the viral inhibitory peptide of TLR4 (VIPER), reduces these effects [44,45].

### 2.3. Structure Modifications and Enzymatic Functions of Electronegative LDL

Electronegativity and apolipoprotein misfolding are two independent features of LDL(−) [46]. The misfolded apoB100 of LDL(−) shows an increased binding affinity to proteoglycans, which may prolong LDL retention in the arterial wall and trigger inflammatory responses [31]. Stabilizing the LDL’s structure through the use of 17-β-estradiol (E2) prevents aggregation; however, it cannot prevent the generation of LDL(−) [46,47]. The structural modifications of apoB100 are associated with phospholipolytic activities and exchange of lipid components [28,48,49]. The sphingomyelinase (SMase)-like activity of LDL(−) may hydrolyze sphingomyelin, which produces apoptotic factor, a ceramide [28,48]. The phospholipase D (PLD) activity of LDL(−) degrades phosphorylcholine, LPC, and sphingomyelin, which is associated with self-aggregation and atherogenic properties. Treatment with 400 μM of chlorpromazine may effectively inhibit both the SMase and PLD activities of LDL(−) [48].

### 2.4. Animal Models Showing Elevated Electronegative LDL

The overproduction of LDL(−) was demonstrated in animal models that consumed a high-fat diet. Lai et al. gave either a standard chow diet or high-fat & high-cholesterol (HFC) diet to each group of 8-week-old male golden Syrian hamsters for six weeks. Plasma LDL-C levels in HFC-diet-fed hamsters were significantly higher than for the control group. Additionally, LDL(−) accounted for 12.5% of all lipoproteins in control hamsters, whereas the value was drastically increased to 42% in HFC-diet-fed hamsters [50]. Recently, Chang et al. distributed an atherogenic diet to sixteen-week-old male New Zealand White rabbits. After six weeks, the LDL(−) from HFC-diet-fed rabbits accounted for about 17.2 ± 5.5% of the LDL fraction. On the other hand, it was almost undetectable in rabbits fed with a control chow diet [51]. Moreover, from the recent publication by Chan et al., LDL(+) and LDL(−) isolated from SLE patients’ LDL samples were then injected into eight-week-old apoE knockout mice. Their results showed that only the LDL(−)-injected mice experienced a significant increase in the plasma CX3CL1 level. By observing histological staining results, LDL(−) can trigger endothelial dysfunction and the formation of atherosclerotic lesions in apoE knockout mice [27]. Taken together, we summarized that LDL(−) plays a vital role in atherosclerosis and plaque formation.

## 3. Mechanisms of Electronegative LDL on Endothelial Cells

The endothelium regulates fluid and molecule trafficking between the bloodstream and tissues for metabolism [52]. In addition, it inhibits platelet aggregation and adhesions by secreting prostacyclin, NO, and exosomes [53,54]. With LDL(−), the atherogenic components lead to endothelial activation and vascular inflammation. Chemokines such as monocyte chemotactic protein-1 (MCP-1) and interleukin-8 (IL-8) are released from the damaged endothelium. The vascular adhesion molecules are highly expressed to promote plaque formation [55]. The mechanisms behind this are listed below.

### 3.1. Phosphatidylinositol-3 Kinase (PI3K)-Serine/Threonine Kinase (Akt) Signaling

The phosphatidylinositol-3 kinase (PI3K)-serine/threonine kinase (Akt) signaling involves the proliferation and survival of endothelial cells through inhibiting pro-apoptotic proteins [56]. Both fibroblast growth factor 2 (FGF2) and vascular endothelial growth factor (VEGF) activate PI3K/Akt signaling [57,58]; in contrast, LDL(−) disrupts Akt phosphorylation, impairing the FGF2 mRNA expression, as well as induces endothelial cell apoptosis [40,59]. In their study, Lu et al. also demonstrated that the apoptotic effects of LDL(−) on endothelial cells could be attenuated by treatment with FGF2 or constitutively expressing active Akt [59]. LDL(−) inhibits B-cell lymphoma 2 (Bcl-2); in contrast, it triggers the expression of Bad/Bax (Bcl-2-associated agonist cell death) and inflammatory factor tumor necrosis factor-α (TNF-α). These actions result in the release of cytochrome c from mitochondria [36,59].

### 3.2. Lectin-Like oxLDL Receptor-1 (LOX-1) Signaling

Lectin-like oxLDL receptor-1 (LOX-1) reacts with multiple ligands in response to danger signals [60]. Patients with cerebral stroke and coronary artery diseases exhibited elevated levels of soluble-form LOX-1 (sLOX-1) [61,62]. Furthermore, patients with ST segment elevation myocardial infarction (STEMI) and rheumatoid arthritis (RA) showed increased sLOX-1 expression in the aspirated coronary thrombi [63,64]. Due to earlier release than biochemical markers of myocardial injury, sLOX-1 could be a novel biomarker for plaque instability [65]. In a hypercholesteremic mice model, the LOX-1 knockout reduced the plaque size and atherosclerotic lesions [66,67,68].

For the detailed mechanisms, LDL(−) leads to the overexpressed changes of LOX-1 on endothelial cells by inducing the expression changes of the pro-inflammatory molecules nuclear factor of kappa light polypeptide gene enhancer in B-cells (NF-κB), vascular cell adhesion molecule (1VCAM-1), and MCP-1 [69,70]. Recently, a similar cohort study was completed to show similar results of LOX-1-mediated inflammation in SLE patients [71]. In addition, the expression of LOX-1 dependents on vasoconstrictors (angiotensin II, endothelin-1) and inflammatory factors such as interferon-γ (IFN-γ), tumor necrosis factor-α (TNF-α), and IL-1β was observed [72]. In vitro, oxidized LDL may enhance the production of angiotensin-converting enzyme (ACE) and endothelin-1 [73,74].

Through LOX-1, LDL(−) downregulates the phosphorylation of Akt and endothelial nitric oxide synthase (eNOS) but increases C-reactive protein (CRP) [11,36,42]. LOX-1 activates Ras homolog family member A (RhoA) and the Ras-related C3 botulinum toxin substrate 1 (Rac1) pathway, leading to the inhibition of intracellular endothelial NO synthesis and overproduction of ROS [75]. Recently, NOS was reported to influence miR-122 expression in hypertension cases, leading to endothelial dysfunction; however, the expression changes of miR-122-mediating endothelial dysfunction remains unanswered. We, therefore, predict LOX-1 signaling of LDL(−) in such cases [76]. Similarly, ROS overproduction leads to p66shc protein phosphorylation, which further deteriorates mitochondrial DNA and contributes to plaque formation [77,78,79]. The phenomenon mentioned above can be attenuated by knocking out the *LOX-1* gene [80,81].

### 3.3. Mitochondria Damage

The basal physiological mechanism of mitochondrial ROS formation is dependent on several factors such as NO, cytosolic Ca2+, and fatty acids [82]. NADPH oxidase 4 (NOX4) in vascular cells inhibits mitochondrial complex I and promotes ROS generation [83]. During the pro-apoptotic conditions, ROS formation is also boosted by growth factor adaptor protein p66Shc, which facilitates the cytochrome c oxidation. Moreover, ROS formation can be further increased by the expression and activation of p66Shc during hyperglycemic conditions [84,85]. LDL(−) inhibits endothelial nitric oxide synthase (eNOS) expression via the Akt signaling pathway, resulting in decreased NO production and leading to endothelial cell apoptosis [86]. Recently, Chen et al. demonstrated that apoE in LDL(−) is responsible for LDL-induced mitochondrial dysfunction. After LDL(−) internalization, apoE translocates from the lysosome to the mitochondria, leading to mitochondrial permeability transition pore (mPTP) opening, dynamin-related protein 1 (DRP1) phosphorylation, and mitochondrial fission [41].

### 3.4. Endoplasmic Reticulum Stress

The intraluminal oxidation in the endoplasmic reticulum (ER) plays a critical role in maintaining calcium concentration and proper folding of transmembrane proteins. The increased amount of lipoprotein promotes a condition known as ER stress, defined by the accumulation of unfolded protein in the ER lumen [87,88]. The molecular mechanism between LDL oxidation and UPR (unfolded protein response)-mediated expression of IL-8, IL-6, and MCP-1 in endothelial cells, which contributes to endothelial dysfunction, is poorly explained [89,90]. Apart from oxidation, glycation of LDL is also found to be a potent marker for dyslipidemia. Studies showed that glycated LDL could initiate nicotinamide adenine dinucleotide phosphate (NADPH) oxidation via ROS production and could induce apoptosis in endothelial cells [91,92]. Therefore, the LDL oxidation and glycation are involved in amplifying endothelial dysfunction and contributing to atherosclerosis.

## 4. Mechanisms of Electronegative LDL on Immune Cells

**Figure 2:**
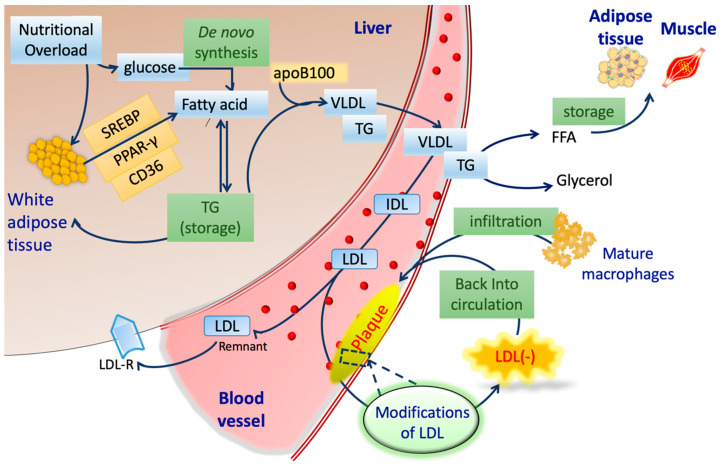
Schematic procedures of lipoprotein metabolism and LDL(−) formation. SREBP: sterol regulatory element-binding protein; PPAR-γ: peroxisome proliferator-activated receptor; CD36: cluster of differentiation 36; TG: triglycerides; apoB100: apolipoprotein B100; VLDL: very low-density lipoprotein; IDL: intermediate-density lipoprotein; LDL: low-density lipoprotein; LDLR: LDL receptor; FFA: free fatty acid.

Alongside endothelial cells, immune cells play a significant role in the pathogenesis of atherosclerosis. Monocytes and T lymphocytes create an inflammatory milieu by releasing several cytokines and growth factors. As LDL(−) concentration is elevated in the blood plasma, it tend to interacts with these monocytes and lymphocytes via cytokines and growth factors [93,94]. LDL(−) impregnates the process of oxidation via the feedback loop mechanism shown in Figure 2 and enhances inflammation. The NEFA and ceramide in LDL(−) also show atherogenic properties [93,95,96,97]. The detailed mechanisms behind this are listed below.

### 4.1. Monocytes

Numerous studies have described the effects of LDL(−) on inducing cytokine release from monocytes, which may be important in atherosclerosis [25,98]. Remodeling of the vascular extracellular matrix (ECM) seemed to be an important landmark of atherosclerosis. LDL(−) induces the release of matrix metalloproteinase (MMP)-9 and tissue inhibitors of metalloproteinase (TIMP)-1 from monocytes through the TLR4/CD14 inflammatory pathway [45]. Additionally, the downstream signal cascade of TLR4/CD14 will then trigger PI3K/Akt signaling and promote p38 mitogen-activated protein kinase (p38 MAPK) phosphorylation, leading to LDL(−)-induced cytokine release from monocytes [99]. The elevated levels of those cytokines may regulate and contribute to vascular plaque formation.

### 4.2. Macrophages

Macrophages play a crucial role in the early stage pathogenesis of atherosclerosis [100]. Circulating monocytes undergo differentiation into macrophages and further polarization into classically activated (M1) or alternatively activated (M2) states in order to withstand environmental stimuli. M1 macrophages are responsible for pro-inflammatory properties, whereas M2 macrophages exert opposing anti-inflammatory properties [101].

According to Yang et al., LDL(+) and LDL(−) isolated from patients with ST segment elevation myocardial infarction (STEMI) were treated with THP-1 macrophages. Their results indicated that only LDL(−) could induce the overproduction of interleukin (IL)-1β [102], granulocyte colony-stimulating factor (G-CSF), and granulocyte–macrophage colony-stimulating factor (GM-CSF) in macrophages through LOX-1-, extracellular signal-regulated kinase (ERK)1/2-, and NF-κB-dependent pathways. Inhibition of ERK1/2 and NF-κB activation can prevent G-CSF and GM-CSF production induced by LDL(−) [103].

In 2020, Chang et al. treated THP-1 with LDL(−), which resulted in increased pro-inflammatory cytokines such as IL-1β, IL-6, IL-8, and TNF-α, as well as M1 surface marker CD86; however, M2-related cytokines and surface marker CD206 were not changed by LDL(−) [39]. Additionally, the expression of CD11c, a marker of M1 macrophages, can also be induced by LDL(−) [104]. LDL(−) can induce M1 polarization of human macrophages responsible for secreting pro-inflammatory cytokines, resulting in foam cell formation and vascular plaque formation.

In addition to human macrophages, in treating LDL(+) and LDL(−) with RAW264.7 cell, the results showed that only LDL(−) can induce the expression of CD95 death receptor (Fas), its ligand CD95 L (FasL), and tumor necrosis factor ligand member 10 (Tnfsf10), which stimulate the activation of the caspases, resulting in cell apoptosis [105].

### 4.3. Platelets

Apart from monocytes and macrophages, accumulating evidence has shown that LDL(−) may trigger platelet activation and aggregation. Platelet hyperreactivity is the most direct evidence contributing to thrombosis in the leading causes of cardiovascular diseases, such as STEMI [106] and stroke [43,107]. As above, Chan et al. separated LDL(+) and LDL(−) from patients with STEMI, with the results illustrating that only LDL(−) was augmented in patients compared to healthy controls. Treating LDL(−) to platelets enhanced their aggregation and adhesion to damaged human aortic endothelial cells (HAECs), which was through LOX-1 and PAFR activation [37]. Furthermore, LDL(−)-induced amyloid β (Aβ) release via IκB kinase 2 (IKK2) in human platelets was reported by Shen et al. in 2016. Besides, LDL(−) works synergistically with Aβ to induce glycoprotein IIb/IIIa receptor activation and phosphorylation of IKK2, IkBa, p65, and c-Jun N-terminal kinase 1 in order to enhance platelet aggregation. These results can be attenuated by inhibiting IKK2, LOX-1, or NF-kB with their inhibitors BMS-345541, TS92, and Bay 117-82, respectively [43]. To conclude, high levels of LDL(−) in patients can trigger platelet activation and aggregation through LOX-1 and PAFR receptors.

## 5. Electronegative LDL in Vascular Diseases

Figure 2 demonstrates the lipid and lipoprotein metabolism in the liver, blood, and peripheral tissues. Nutritional overload increases fatty acids via the overexpression of cluster of differentiation 36 (CD36) and peroxisome proliferator-activated receptor (PPAR-γ) [108,109,110]. This phenomenon is highly contrasted to the de novo synthesis pathway, although FFAs from either source in the liver are indistinguishable. The elevated level of free fatty acids ultimately increases triglyceride through esterification. Combined with apoB100 and triglyceride, the efflux of VLDL into circulation promotes the pro-atherogenic metabolic state. VLDL particles deliver lipids hydrolyzed by lipoprotein lipase (LPL) and release FFAs in plasma [111,112,113].

With the increasing incidence of LDL retention in endothelial cells [114,115,116,117,118], the LDL particles reportedly undergo oxidative modifications by macrophages and endothelial cells within arterial walls (Figure 2) [119,120,121,122,123,124]. The accumulation of oxLDL further boosts the electronegativity, ultimately generating LDL(−) in circulation [33]. LDL(−) is highly atherogenic and pro-apoptotic to the vascular system, including the endothelium of the blood–brain barrier (BBB). Wang et al. in 2017 explored the role of LDL(−) in pheochromocytoma-derived cell line (PC12) cells, where deliberate dosages of LDL(−) induced neurotoxic stress in a LOX-1-dependent manner [125].

The presence of LDL(−) in circulation correlates with atherosclerosis progression and endothelial dysfunction-mediated cardiovascular diseases. LDL(−) levels are significantly higher in frequent smokers, diabetic patients, and hypercholesterolemia patients [33,34,40,59]. In addition, LDL(−) levels were 10-times higher in STEMI and stroke patients, even though the LDL-C levels were similar to healthy controls [37,43].

## 6. Current Treatment Strategies Targeting Electronegative LDL

The diagnosis and treatment for endothelial damage are dependent on the ankle–brachial index, vascular imaging, surgery, and revascularization [126,127,128]. Currently, treatment for dyslipidemia and the prevention of microvasculature damage mainly revolve around reducing LDL-C levels [129,130,131]. A plethora of studies have demonstrated that excessive levels of lipids lead to endothelial damage; however, only a few studies have outlined strong mechanistic interactions between lipid alterations and endothelial dysfunction (Table 1).

Statins, the inhibitors of β-hydroxy β-methylglutaryl-CoA (HMG-CoA), are successful in lowering cholesterol loadings and expression of LOX-1; they also inhibit atherosclerotic progression and acute atherothrombosis [162,163,164]. Additionally, statins effectively reduce the proportion of LDL(−) [165,166,167,168]; discontinuation leads to LDL(−) approaching baseline levels [42]. However, the mechanisms of LDL(−) reduction are still not clear. Ezetimibe inhibits the Niemann–Pick C1-like 1 transporter (NPC1L1), which leads to decreased cholesterol absorption [169]. Proprotein convertase subtilisin kexin type 9 (PCSK9) is an enzyme for the degradation of LDL receptor (LDLR); blocking PCSK9 may increase LDLR, therefore lowering blood LDL-C concentrations. PCSK9 inhibitors such as alirocumab and evolocumab aggressively reduce the degradation of LDL receptors and increase the clearance of LDL cholesterol in hepatic cells [170]. They increase plaque stability but decrease the necrotic lipid core, as shown in Figure 1 [171,172,173,174,175]. However, other than statins, whether these drugs can decrease LDL(−) or not is currently unclear.

Several anti-inflammatory approaches were taken here to study the management of dyslipidemia, such as cell therapy using mesenchymal stem cells [176], leukotriene inhibitors [177], chemokine ligands (CC motif ligand), MCP-1, IL-1, and TNF-α blockers for the prevention of atherosclerotic plaque formation [178,179,180,181,182,183,184]. The currently used drugs significantly decrease LDL-C levels, stabilize vascular plaque, and slowdown atherosclerotic progression; however, new therapeutic strategies for LDL(−) and biomarkers are still needed.

## 7. Perspective

LDL(−) plays a critical role in the pathophysiology of atherogenesis. It triggers the dysfunction of endothelium by macrophage differentiation, monocyte migration, and platelet aggregation. Moreover, LDL(−) impairs endothelial cells by superoxide overproduction and platelet activation [185,186,187]. In combination with other well-known markers of inflammation, namely metabolic diseases, we concluded that LDL(−) can be a novel prognostic tool for these lipid disorders. Regarding treatment for the prevention of ASCVD, even though statins can partially reduce the concentration, finding a way to clear LDL(−) remains of utmost importance [22]. In particular, a method involving hydrolyzing atherogenic lipids in LDL(−) and producing harmless metabolites might be a novel therapeutic approach in the future.

## Figures and Tables

**Table 1:** Primary dyslipidemia markers and pathways involved in different diseases.

Diseases	Dyslipidemia Markers	Drug Treatment	Effect on ED	Pathway/Phenomenon Involved	Studied on	References
Hypertension	NOS, ROS	α-Linolenic acid	Yes	SIRT-3	Mice	[132]
Hypertension	NOS, ROS	____	Yes	miR-122, CAT-1	Human	[76]
Hypertension, Angina	NOS, CRP, Hyperglycemia	Carvedilol	Yes	β-adrenergic mediate Vasodilation	Human	[133,134,135,136]
Heart failure	oxLDL, LDL	Rosuvastatin	Yes	Inflammatory markers	Human	[137,138]
ACS	oxLDL, LDL-C and cardiac fibrosis	perindopril	Yes	--	Human	[139,140]
CKD, CHF	Cardiac fibrosis	carvedilol	Yes	β-adrenergic mediate Vasodilation	Human	[141]
LVF, CKD	oxLDL, LDL and Cardiac fibrosis	Renal and heart transplant	-----	-----	Human	[142]
STEMI	____	Enoxaparin, Clopidogrel and β-blocker	No	Case study	Human	[143]
STEMI	Atherosclerotic Plaques	Statins, Aspirins, β-blocker, ACE-inhibitor	Yes	___	Human	[144,145]
STEMI	CRP and Atherosclerotic plaques	Vit B, B6, and B12	No	Homocysteine	Human	[146]
STEMI	LDL-C, Ox-LDL and L5	___	___	PKC/AKT pathway	Mice	[37]
CAD, Diabetes	NOS, Hyperinsulinemia, Hyperglycemia	Pioglitazone	Yes	Anti-inflammation, Vasodialation	Human	[147,148]
T1DM	Cardiac fibrosis	Fingolimod (FTY720)	Yes	Rag-1	Mice	[149]
T2DM	Hyperglycemia and Cardiac Fibrosis	H2/H3- RLX	Yes	α-SMA, MMP, TIMP and NLRP3	Rat	[150,151]
T2DM	NOS and Hyperglycemia	Berberine	Yes	AMPK and eNOS Phosphorylation	In-vitro, Ex-vivo	[152,153,154]
T2DM	Hyperglycemia, oxLDL, LDL, TG	Fenofibrate	Yes	PPAR-α/γ	Rat	[155,156]
RA	CRP, LDL, TG	MTX and Glucocorticoid	Yes	Hemodynamics	Human	[157]
RA	NOS, Myeloperoxidase, LDL	Tocilizimab	Yes	JAK/STAT and mTOR	Human	[158]
Stroke SLE	Atherosclerotic plaques	Glucocorticoids, Immunosuppressant	Yes	-----	Human	[159,160]
SLE	Atherosclerotic plaques	Anifrolumab and tsDMARDs	Yes	JAK/BTK	Human Phase III	[161]

RP: C-reactive protein; LDL-C: Low-density lipoprotein cholesterol; TG: Triglyceride; MTX: Methotrexate; RA: Rheumatoid arthritis; NHC: Normal healthy control; T1DM: Type 1 diabetes mellitus; Rag-1: Recombination-activating gene 1; NOS: Nitric oxide synthase; JAK/STAT: Janus kinase/signal transducer activator of transcription protein; T2DM: Type 2 diabetes mellitus; H2/H3-RLX: Relaxin-1 and Relaxin3; mTOR: mammalian target of rapamycin; α-SMA: Alpha smooth muscle actin; MMP: Matrix metallopeptidase; TIMP: Tissue inhibitor of metalloproteinase; NLRP3: NOD-LRR and pyrin-domain-containing protein 3; Ox-LDL: oxidized low-density lipoprotein, CHF: Chronic heart failure; CVD: Cardiovascular disease; AMPK: AMP (Adenosine monophosphate)-activated protein kinase; eNOS: endothelial NOS; PPAR-γ/α: Peroxisome proliferator-activated receptor alpha/gamma; CKD: Chorionic kidney disease; LVF: Left ventricular failure; SLE: Systemic lupus erythematosus; tsDMARDs: Targeted synthetic disease-modifying antirheumatic drugs; JAK/BTK: JAK/Bruton’s tyrosine kinase (inhibitor); STEMI: St-elevation myocardial infarction; ACE: Angiotensin-converting enzyme; ROS: Reactive oxygen species; SIRT-3: Nicotinamide adenosine diphosphate (NAD)-dependent deacetylase sirtuin-3; miR122: MicroRNA 122; CAT-1: Cationic amino acid transporter 1; PKC/AKT: Protein kinase C/protein kinase B.

## Abbreviations

Aβ	Amyloid β
ACE	Angiotensin-converting enzyme
ADP	Adenosine diphosphate
ADPase	Ecto-Adenosine diphosphate
ApoB100	Apolipoprotein B100
ApoCIII	Apolipoprotein CIII
ApoE	Apolipoprotein E
ASCVD	Atherosclerotic cardiovascular diseases
Bad/Bax	BCL2-associated agonist of cell death
Bcl-2	B-cell lymphoma 2
BBB	Blood–brain barrier
BP	Blood pressure
CAD	Coronary artery disease
CCL	Chemokine ligand
CD	Cluster of differentiation
CER	Ceramide
cIMTPWV	Carotid intermedia thickness and pulse wave velocity
COX	Cyclooxygenase
CD36	Cluster of differentiation 36
CRP	C-reactive protein
CVD	Cardiovascular disease
EC	Endothelial cell
ECM	Extracellular matrix
ED	Endothelial dysfunction
ERK	Extracellular signal-regulated kinase
eNOS	Endothelial nitric oxide synthase
ER	Endoplasmic reticulum
Fas	CD95 death receptor
FasL	Ligand CD95 L
FFA	Free fatty acids
FGF2	Fibroblast growth factor 2
FPLC	Fast-protein liquid chromatography
G-CSF	Granulocyte colony-stimulating factor
GDF	Growth differentiation factor
GM-CSF	Granulocyte–macrophage colony-stimulating factor
HDL	High-density lipoprotein
HFC	High-fat, high-cholesterol
HIF-1α	Hypoxia-inducible factor-1α
HMGCoA	β-hydroxy β-methylglutaryl-CoA
HUVECs	Human umbilical vein endothelial cells
ICAM	Intracellular adhesion molecule 1
IDL	Intermediate-density lipoprotein
IFN-γ	Interferon- γ
IKK2	IκB kinase 2
IL	Interleukin
iNOS	Inducible NO synthase
IR	Insulin resistance
IRAK2	Interleukin-1 receptor-associated kinase-2
IRE-1	Inositol requiring enzyme-1
Lp(a)	Lipoprotein (a)
LDL	Low-density lipoprotein
LDL(−)	Electronegative LDL
LDL-C	LDL cholesterol
LPC	Lysophosphatidylcholine
LPL	Lipoprotein lipase
LOX-1	Lectin-like oxidized low-density lipoprotein receptor-1
MAPK	Mitogen-activated protein kinase
MCP-1	Monocyte chemotactic protein-1
MetS	Metabolic syndrome
MMP	Metalloproteinase
MSC	Mesenchymal stem cell
NADPH	Nicotinamide adenine dinucleotide phosphate
NEFA	Non-esterified fatty acids
NF-κB	Nuclear factor kappa light-chain enhancer of activated B cells
NO	Nitric oxide
Nox	NADPH oxidase
NPC1L1	Niemann–pick C1-like
oxLDL	Oxidized LDL
PAFR	Platelet activating factor
PAFR	Platelet activating factor receptor
PC12	Pheochromocytoma cell-derived cell line
PGI2	Prostacyclin 2
PI3K	Phosphatidylinositol-3 kinase
PLD	Phospholipase D
PSCK9	Proprotein convertase subtilisin kexin type 9
RhoA	Ras homology family member A
Rac1	Ras-related C3 botulinum toxin substrate 1
Smase	Sphingomyelinase
STEMI	ST segment elevation myocardial infarction
TGF-β	Transforming growth factor- β
TIMP	Tissue inhibitors of metalloproteinase
TLR4	Toll-like receptor 4
TNF-α	Tumor necrosis factor-α
Tnfsf10	Tumor necrosis factor ligand, member 10
UCP 2	Uncoupling protein 2
UPR	Unfolded protein response
VCAM-1	Vascular cell adhesion molecule-1
VEGF	Vascular endothelial growth factor
VIPER	Viral inhibitory peptide of TLR4
VLDL	Very low-density lipoprotein
VSMCs	Vascular smooth muscle cells
